# Tsunamigenic potential of flank instabilities of Kameni Volcano inside Santorini Caldera, southern Aegean

**DOI:** 10.1007/s00531-026-02623-5

**Published:** 2026-10-21

**Authors:** B. Bauer, J. Preine, P. Nomikou, T. Kwasnitschka, M. Blanch Jover, J. Karstens, F. Gross, L. Rüpke, H. Kopp

**Affiliations:** 1 GEOMAR Helmholtz Centre for Ocean Research Kiel, Kiel, Germany; 2 Marine Geoscience, National Oceanography Centre, Southampton, UK; 3 Department of Geology and Geoenvironment, National and Kapodistrian University of Athens, Athens, Greece; 4 Center for Ocean and Society, Kiel University, Kiel, Germany

**Keywords:** Landslide-HySEA code, Santorini volcanic complex, Kameni volcano, Numerical tsunami modeling, Seafloor microbathymetry

## Abstract

Located in the southern Aegean Sea, the Santorini volcanic complex represents one of the most geodynamically active and hazardous volcano–tectonic systems in the Mediterranean. Following the Late Bronze Age caldera-forming eruption (~ 1600 BCE), the Kameni volcanic edifice emerged within the central caldera. On the northwestern flank of Nea Kameni, lava flows are superposed on unconsolidated pyroclastic deposits, primarily pumice and ash attributed to the 726 CE submarine explosive eruption. These units reside on a topographic step coincident with a major volcano–tectonic lineament, a geomorphological configuration that exhibits structural parallels to the pre−2018 sector collapse state of Anak Krakatau. Here, we employ the Landslide-HySEA numerical code to simulate tsunami generation and propagation resulting from various potential flank instability scenarios of Nea Kameni. Three distinct collapse volumes were modeled: 0.057 km^3^, 0.077 km^3^, and 0.13 km^3^. The simulations demonstrate substantial hazardous potential; a flank failure of 0.13 km^3^ has the potential to generate maximum coastal wave amplitudes of up to 39 m. Furthermore, all modeled scenarios result in tsunami heights exceeding 2 m at multiple critical coastal locations. These results indicate that such events would inflict severe damage on critical infrastructure and high-density maritime hubs situated along the inner caldera cliffs. Seafloor microbathymetry acquired through a photogrammetric tow-sled system suggests little remobilization despite ongoing volcano–seismic activity. Yet, our findings underscore the necessity for continuous monitoring of the structural stability of the Kameni volcanic edifice and emphasize the requirement to integrate flank-collapse mechanisms into the tsunami hazard assessment frameworks for Santorini.

**Graphical abstract:**

**Figure:**
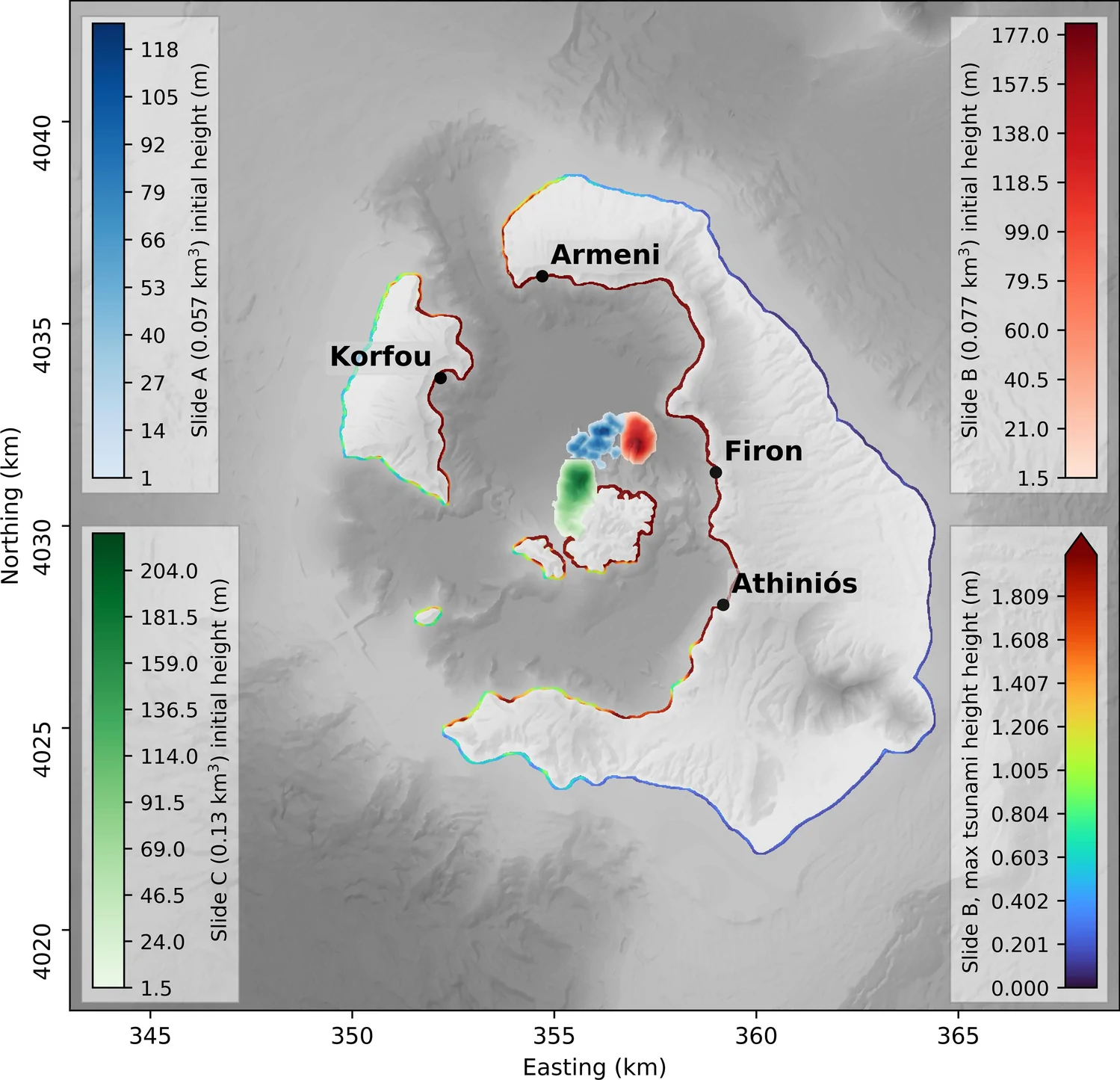

**Supplementary Information:**

The online version contains supplementary material available at https://doi.org/10.1007/s00531-026-02623-5.

## Introduction

Tsunamis generated by volcanic island sector collapses present a recognized threat to coastal communities and infrastructure. In historical times, 18 documented sector collapse events have caused more than 15 000 casualties (Siebert et al. 1987, 2006; Auker et al. 2013; Day 2015). With a volume of ~2.4 km^3^, the 1888 Ritter Island sector collapse is considered to be the most voluminous sector collapse in historical times and triggered a tsunami that reached settlements up to 600 km away with maximum tsunami heights of more than 20 m on neighboring islands of Papua New Guinea (Johnson 1987; Ward and Day 2003; Karstens et al. 2019). However, even much smaller events have the potential to trigger impactful tsunamis. With a volume of 0.2–0.3 km^3^, the 2018 volcanic sector collapse of Anak Krakatau, located in the Sunda Strait of Indonesia, triggered a tsunami with up to 13 m run-up heights along the surrounding coasts of Sumatra and Java, which caused more than 400 casualties (Walter et al. 2019; Grilli et al. 2019; Cutler et al. 2022). The 0.02 km^3^ collapse at Stromboli in 2002 triggered a tsunami that caused destruction at the island’s coastal settlements with run-up heights of up to 10 m (Bonaccorso et al. 2003; Maramai et al. 2005; Tinti et al. 2006; Di Traglia et al. 2023).

**Fig. 1:**
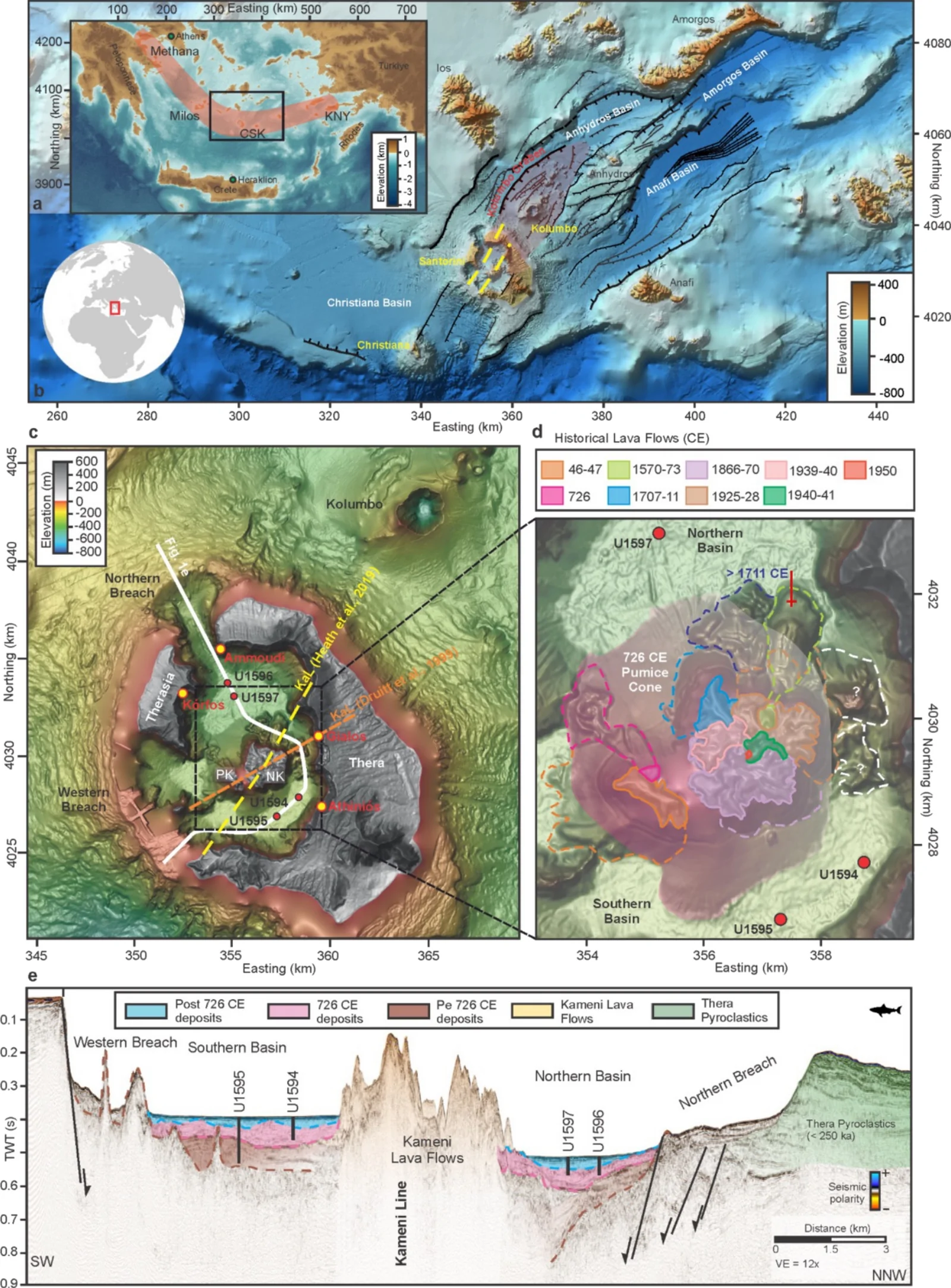
**a** Regional map of the southern Aegean showing the South Aegean Volcanic Arc (shaded red) and the study area (black rectangle). Major cities of the Aegean coastline are indicated in green. The coordinate system here and in other maps in this panel is UTM Zone 35N, WGS84 datum. CSK: Christiana–Santorini–Kolumbo. KNY: Kos–Nisyros–Yali. **b** Morphological map of the Christiana–Santorini–Kolumbo Volcanic Field and the Santorini–Amorgos Tectonic Zone. Major basin-bounding faults are indicated by thick lines (Nomikou et al. 2018; Preine et al. 2026) minor faults are illustrated in black (modified from Nomikou et al. (2018), Isken et al. (2025), and Preine et al. (2026)). The red line indicates the Kolumbo Fault (KF). Semi-transparent green area indicates the extent of the Kolumbo Graben in the western Anhydros Basin. Yellow dashed lines indicate the Kolumbo and Kameni lines (Druitt et al. 1999; Heath et al. 2019). Semi-transparent area indicates the Kolumbo Graben (Preine et al. 2026). **c** Morphological map of the Santorini caldera showing IODP Expedition 398 drill sites (red circles), as well as coastal villages within the inner caldera (yellow circles). Orange dashed line indicates the Kameni Line (KaL) as defined by Druitt et al. (1999) by the linear alignment of post-Monian vents. Yellow dashed line indicates the Kameni Line as defined by Heath et al. (2019) from seismic P-wave tomography. *NK* Nea Kameni, *PK* Palea Kameni (modified from Preine et al. (2024)). White line indicates the location of the seismic line shown in panel **e**. **d** Enlargement from c highlighting the Kameni Volcano with dated onshore lava flows bounded by solid lines and shown in semi-transparent colors (Pyle et al. 2006; Preine et al. 2024). The offshore continuation of lava flows are marked by dashed lines (Nomikou et al. 2014a, b; Preine et al. 2024). The pink semi-transparent area indicates the outline of where the pumice cone from the 726 CE eruption of Kameni is inferred to crop out at the seafloor (Preine et al. 2024). White dashed lines indicate lava flows of unknown ages. Red line indicates the MOMO transect. **e** Seismic profile crossing the caldera of Santorini. Location is shown in Fig. 1c. TWT: Two-way travel time; VE: vertical exaggeration. Semi-transparent colors indicate seismically resolved deposits within and outside the caldera (Preine et al. 2022a, b, 2024)

Located in the central Aegean Sea (Fig. 1a, b), Santorini is one of the most intensively visited islands in the Mediterranean, with approximately 2 million visitors annually (Peeters et al. 2018). Over the past decade (2016–2023), tourism pressure has increased markedly, reflected in rising visitor numbers, hotel capacity, and the expansion of built-up areas; a trend that is projected to continue and is associated with significant environmental, social, cultural, and economic impacts (Leka et al. 2025). The island’s permanent population is officially recorded at 15,550 (Hellenic Statistical Authority 2025). During the summer season, this population is substantially augmented by temporary workers employed in the tourism sector.

This highly dynamic population exposure coincides with a complex multi-hazard volcanic setting, where hazard assessment plays a critical role in communicating risks to a wide range of stakeholders (Charlton et al. 2020). While hazard and impact assessments related to volcanic eruptions are widely used by disaster risk reduction agencies and civil protection authorities, non-eruptive volcanic hazards, particularly those associated with episodes of volcanic unrest, remain comparatively underrepresented (Charlton et al. 2020; Hayes et al. 2020; Kaye et al. 2009; Selva et al. 2019, 2020; Zuccaro et al. 2008). Periods of unrest, such as the 2011–2012 Santorini episode (Parks et al. 2012) or the 2025 earthquake swarm (Isken et al. 2025), can enhance the likelihood of cascading hazards, including tsunamis triggered by submarine landslides promoted by deformation, seismicity, and hydrothermal alteration (Selva et al. 2020). Such unrest episodes occur annually at approximately 20 large calderas worldwide and may persist for years, often with considerable socio-economic consequences (Acocella et al. 2015; Charlton et al. 2020; Newhall and Dzurisin 1855; Phillipson et al. 2013; Potter et al. 2015; Barberi et al. 1984; Johnston et al. 2002; Longo 2019). Moreover, even moderate-scale events can produce catastrophic impacts due to the concentration of critical infrastructure in active volcanic regions (Mani et al. 2021).

Within Santorini volcanic group, susceptibility maps addressing volcanic activity and related processes, such as landslides (Forte et al. 2019), ashfall, and gas hazards (Jenkins et al. 2015), as well as numerical modeling of tsunami scenarios (Katsigera et al. 2024; Necmioglu et al. 2023; Novikova et al. 2011) are available (General Secretariat for Civil Protection 2023). However, focused assessments of present-day tsunami hazard remain limited. Building vulnerability analysis in the town of Kamari on the southwestern shore of Santorini based on a worst-case tsunami run-up scenario of 10 m above sea level (caused by an earthquake with Mw ~8.5) suggested that more than 400 structures would fall within the inundation zone, with over half classified as highly to very highly vulnerable (Batzakis et al. 2020). Seismic studies of the Kolumbo submarine volcano further indicate that submarine slope failures associated with volcanic or volcano-triggered seismic activity could generate tsunamis, where even relatively low-magnitude explosive events may pose a substantial risk due to the volcano’s proximity to Santorini’s eastern coastline (Nomikou et al. 2014a; Karstens et al. 2023a, b; Tadini et al. 2025). Accordingly, secondary seismic and volcanic effects, including slope instabilities, rockfalls, and tsunamis have been highlighted as essential components of future multi-hazard analyses (Kazantzidou-Firtinidou et al. 2018).

In addition to the previously known tsunami hazard sources, recent results from IODP Expedition 398 point to a previously overlooked hazard scenario for Santorini. Between December 2022 and February 2023, IODP Expedition 398 recovered several 10 s of meters of unconsolidated pumice and ash at four drill sites within Santorini Caldera (Preine et al. 2024; Druitt et al. 2024a, b). These volcaniclastic deposits were emplaced during a submarine explosive eruption of Kameni Volcano in 726 CE (Preine et al. 2024). The eruption produced pumice and ash with a bulk volume of up to 3.1 km^3^ centered around Kameni Volcano (Fig. 1c, d) (Preine et al. 2024). Seven subsequent eruptions of Kameni Volcano formed thick lava flows on top of the up to 20º steep slopes of underconsolidated pumice and ash deposits of the 726 eruption (Preine et al. 2024). Owing to the position of the Kameni Volcano on top of a topographic step coincident with the volcano–tectonic Kameni Line, previous research has identified the edifice as potentially susceptible to flank instability, necessitating the inclusion of flank collapse in regional hazard assessments (Preine et al. 2024). Despite these indications, this specific mechanism has hitherto remained unquantified and has not yet been integrated into existing disaster risk frameworks.

Necmioglu et al. (2023) presented a first-order quantification for the tsunamigenic potential of three possible landslide scenarios on Santorini (i.e., volumes of up to 0.135 km^3^). In their study, the dynamic generation phase of the landslide is simplified via empirical equations and the initial waves are approximated as a Gaussian shape with maximum initial wave amplitude initial length of the sea-surface displacement. Here, we present complementary first-order quantification for the tsunamigenic potential within the caldera of Santorini by investigating if a previously underappreciated flank-collapse mechanism of Kameni Volcano (i.e., lava flows emplaced onto unconsolidated 726 CE deposits) can generate hazardous tsunamis. Our study has the following objectives: (1) To provide a first-order qualitative assessment for tsunamis resulting from flank collapses of different volumes (i.e., 0.057 km^3^, 0.077 km^3^, and 0.13 km^3^) through numerical modeling. (2) To assess exposure and vulnerability of maritime infrastructure within the Santorini caldera. (3) To investigate flow emplacement and possible remobilization through seafloor microbathymetry acquired by a photogrammetric tow-sled system.

## Geological setting

The Christiana–Santorini–Kolumbo volcanic field forms the central segment of the South Aegean Hellenic Volcanic Arc (Nomikou et al. 2019), which developed in response to oblique subduction of the African plate beneath Eurasia (Fig. 1a, b) (Le Pichon and Angelier 1979; Royden and Papanikolaou 2011). Since the Late Miocene, slab rollback, crustal collapse, westward Anatolian motion, and slab tearing have driven southward retreat of the arc and progressive extension within the Aegean microplate (Jolivet 2001; Le Pichon and Kreemer 2010). These processes produced the horst-and-graben morphology of the Cyclades and thinned continental crust (20–30 km), reflecting long-lived polyphase deformation (Zhu et al. 2006).

Early N–S extension generated E–W striking fault systems and basin subsidence (Anastasakis and Piper 2005), while increasing arc curvature during the Pliocene–Pleistocene introduced NE–SW striking structures accommodating block rotations (Gautier et al. 1999; Van Hinsbergen and Schmidt 2012). Among these is the Santorini–Amorgos Tectonic Zone (SATZ), a major NE–SW oriented rift system composed of en-echelon basins, such as the Anhydros, Anafi, and Amorgos basins, containing >1 km of sediment and bounded by large extensional faults (Fig. 1b) (Nomikou et al. 2018; Preine et al. 2020). Within this extensional framework, the Christiana–Santorini–Kolumbo volcanic field has been active since ~3–4 Ma (Nomikou et al. 2013). It comprises Christiana, Santorini, Kolumbo, and the Kolumbo Volcanic Chain (Nomikou et al. 2012), located at the intersection of the Christiana and Anhydros basins (Preine et al. 2022a). Recent IODP Expedition 398 results show that Christiana Volcano is significantly older (2.5–2.7 Ma) and predates Santorini–Kolumbo activity (Vroon et al. 2025).

Santorini’s evolution is divided into Ancient Santorini (> 825–570 ka), dominated by rhyolitic volcanism, and Modern Santorini (< 570 ka), characterized by basalt to rhyodacite compositions (Metcalfe et al. 2025b). A major early explosive event, the Archaeos eruption (~ 765 ka), forms a regional stratigraphic marker (Preine et al. 2022a, b; Druitt et al. 2024a, b). Between 570 and 250 ka, activity was largely effusive to moderately explosive. Around 250 ka, the system transitioned to highly explosive caldera-forming silicic eruptions, producing the Thera Pyroclastic Formation and four major caldera collapses, including the Late Bronze Age Minoan eruption (~ 3.6 ka), one of the largest Holocene eruptions (Druitt et al. 1999; Kutterolf et al. 2021; Karstens et al. 2023c). Volcanism during this phase was focused in the northern caldera basin above a shallow magma reservoir bounded by the Kameni and Kolumbo lineaments (Heath et al. 2019; Hooft et al. 2017).

Kolumbo volcano, ~7 km northeast of Santorini, is fed by a shallow felsic reservoir at 2–5 km depth linked to a mid-crustal melt body at ~11–13 km depth (Fig. 1b, c) (Chrapkiewicz et al. 2022; Autumn et al. 2025). Seismic and tephra records indicate at least 19 eruptions since ~265 ka, with the most recent in 1650 CE (Karstens et al. 2023a, b; Metcalfe et al. 2026).

The volcanic evolution of Santorini and Kolumbo appears closely coupled to regional tectonics. The onset of caldera-forming explosive activity around 250 ka broadly coincides with major changes in basin subsidence within the SATZ, expressed by regional onlap surfaces (Preine et al. 2022a, b; Metcalfe et al. 2025b). While internal magmatic evolution may have contributed, accelerated rifting and enhanced melt flux are increasingly favored as drivers of this transition (Metcalfe et al. 2025a). All volcanic vents within the last 250 ka clustered within the Kolumbo Graben, which is geophysically imaged in the western Anhydros Basin and inferred to continue underneath present-day Santorini (Fig. 1b) (Preine et al. 2026).

The Christiana–Santorini–Kolumbo volcanic field is therefore one of the most active and hazardous volcano–tectonic systems in the Mediterranean, with a long history of large earthquakes, caldera-forming eruptions, landslides, sector collapses, and tsunami generation (Druitt et al. 1999, 2019; Bell et al. 2013; Nomikou et al. 2019; Karstens et al. 2023a, b). Historical tsunamis include those associated with the Minoan eruption and the 1650 CE Kolumbo eruption (Dominey-Howes et al. 2000; Ulvrová et al. 2014, 2016; Paris et al. 2022). Recent unrest episodes, including inflation at Santorini in 2011–2012 and the 2025 Anhydros Ridge seismic swarm linked to dike intrusion from Kolumbo’s mid-crustal magma storage (Isken et al. 2025; Lomax et al. 2025), highlight the ongoing dynamic coupling between tectonics and magmatism.

## Potential Kameni flank-collapse scenarios

Kameni Volcano, located in the center of Santorini’s caldera, represents the post-collapse intra-caldera edifice formed following the Late Bronze Age ‘Minoan’ caldera-forming eruption (~ 1600 BCE), marking the onset of a new caldera cycle (Fig. 1c, d) (Druitt et al. 1999; Pyle et al. 2006; Nomikou et al. 2014b; Watts et al. 2015). Similar to other multi-cyclic silicic caldera systems, Santorini’s post-caldera evolution has been characterized by extended periods of effusive to mildly explosive volcanism, during which Kameni Volcano progressively built up as a largely submarine edifice with two emergent summits, Palea Kameni and Nea Kameni (Druitt et al. 1999; Nomikou et al. 2014b). Historical records document multiple eruptions since at least 197 BCE, with activity continuing until 1950 CE, followed by a phase of volcanic unrest in 2011–2012 (Fouqué, 1879; Druitt et al. 1999; Pyle et al. 2006; Parks et al. 2012).

High-resolution seismic reflection data integrated with results from IODP Expedition 398 reveal that much of the Kameni edifice, particularly its submarine flanks, is constructed upon thick accumulations of unconsolidated pumice and ash deposits emplaced during a major explosive eruption in 726 CE (Fig. 1d, e) (Preine et al. 2024). These deposits form steep slopes of up to ~20° along a pronounced topographic step separating the northern caldera basin (maximum depth: 385 m) from the southern basin (maximum depth: 290 m). The step is aligned with the Kameni Line, an active volcano–tectonic lineament (Fig. 1c–e) (Druitt et al. 1999; Heath et al. 2019; Nomikou et al. 2014b). Recovered deposits of the 726 CE eruption consist of upward-grading pumice lapilli to lapilli-ash with pumice clasts (Preine et al. 2024). Subsequent lava flows since 1570–1573 CE were emplaced directly onto these weak, poorly consolidated volcaniclastic units, creating mechanically unstable conditions within the growing edifice (Fig. 1d, e).

Such a configuration represents a well-known preconditioning factor for flank collapse at marine volcanoes (Siebert 1984; Van Wyk de Vries and Francis 1997; Keating and McGuire 2000; Furst et al. 2023), where steep volcanic slopes underlain by unconsolidated deposits are particularly susceptible to failure, especially during periods of magma intrusion, seismicity, or hydrothermal alteration. Comparable conditions preceded the catastrophic 2018 sector collapse of Anak Krakatau (Giachetti et al. 2012; Grilli et al. 2019). At Kameni, flank instability may be further enhanced by its position at the edge of a deep intra-caldera depression and its proximity to zones of past ground deformation associated with magma recharge beneath the caldera (McVey et al. 2019).

Collectively, the rapid post-caldera growth of the Kameni volcanic edifice, the superposition of lava flows onto steep, unconsolidated pyroclastic deposits, the structural influence of the Kameni Line, and recurrent episodes of volcanic unrest establish the fundamental preconditions for potential flank instability. A future flank failure of the Kameni Volcano, paralleling documented gravitational collapses at analogous submarine and littoral volcanic systems, possesses the capacity to generate tsunamis both within the Santorini caldera and across the broader region. Consequently, these mechanisms warrant formal integration into regional geohazard and risk-mitigation frameworks.

**Fig. 2:**
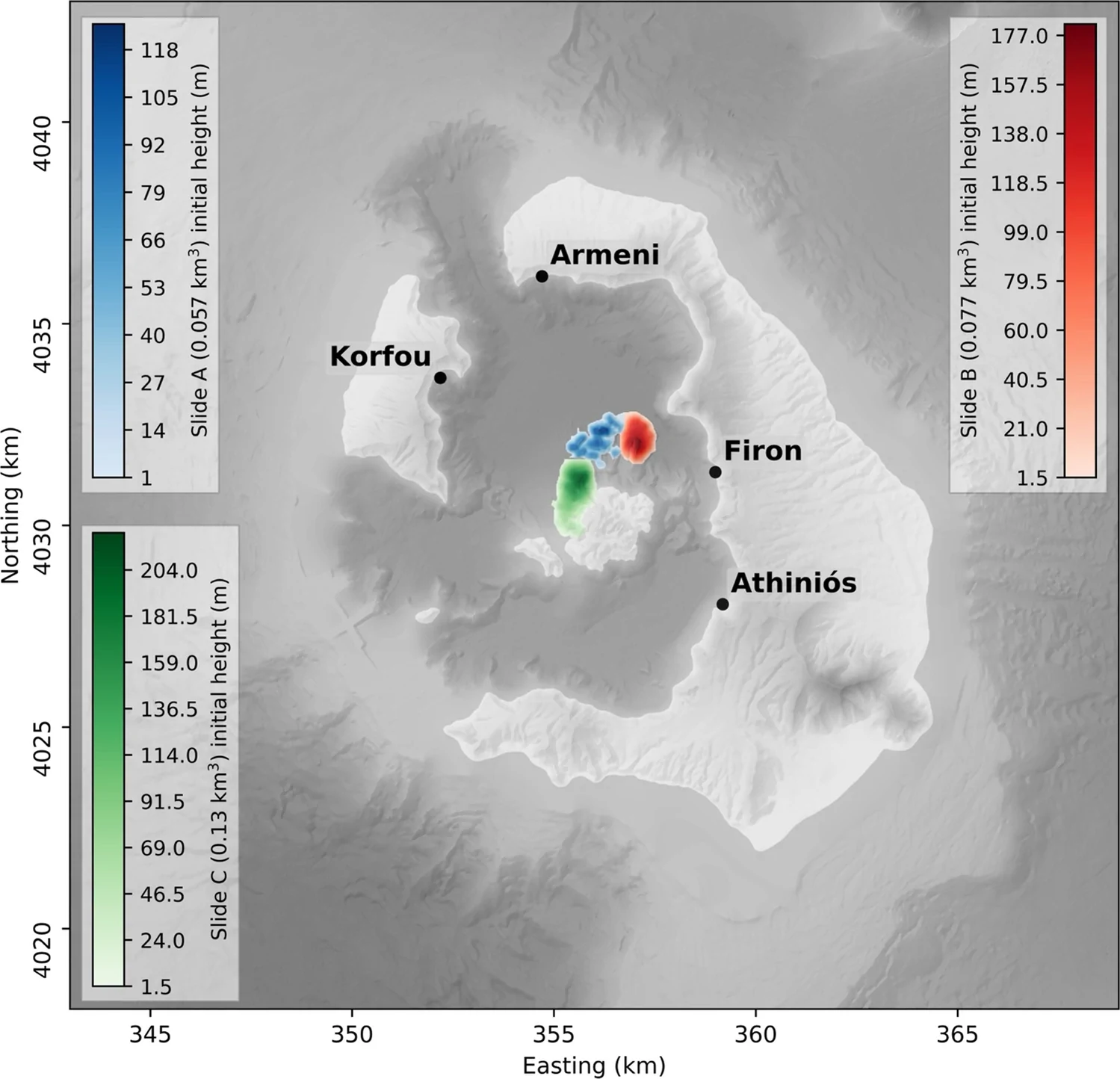
Potential slide layers. Overview of modeling area and initial slide thickness for each scenario (A=blue, B=red, C=green). Locations for further analysis are indicated as black dots (i.e., Korfou, Armeni, Firon, Athiniós)

Three different flank-collapse scenarios at Kameni Volcano are considered in this study, in the following referred to as slides A, B, and C (blue, red, and green in Fig. 2, respectively). As shown in Fig. 2, slide A includes multiple lava flows between 1711 and 1925 CE with a total volume of 0.057 km^3^, slide B consists of 0.077 km^3^ of the 1570–1573 CE lava flow, and slide C corresponds to the 1707–1711 CE lava flow with a volume of 0.13 km^3^ (Fig. 1d) (Preine et al. 2024; Druitt et al. 2024a, b). These scenarios were chosen as a first-order approximation, to cover a reasonable range of slide volume and orientation.

## Methods

### Landslide HySEA code

The depth-averaged Landslide-HySEA (L-HySEA) code was developed by the EDANYA group at the University of Malaga for numerical modeling of tsunamis generated by submarine and aerial landslides. The finite-volume numerical model is based on the two-layer Savage–Hutter model (Savage and Hutter 1989) described by Fernández-Nieto et al. (2008) and was fully validated for all NOAA’s National Tsunami Mitigation Program mandatory benchmark problems (Macías et al. 2020a, 2017a, 2020b, 2017b), as well as applied to field cases (González-Vida et al. 2019; Macías et al. 2015). System equations are described in detail by Macías et al. (2015) and González-Vida et al. (2019). Following Fernández-Nieto et al. (2011), the discretization of system equations is done with an explicit first-order IFCP (Intermediate Field Capturing Parabola). The discretization of the Coulomb friction law is done as presented by Fernández-Nieto et al. (2008). The treatment of wet–dry fronts is implemented as proposed by Castro et al. (2005). The numerical scheme is implemented on GPUs using the CUDA programming language, as described by Castro et al. (2011) and de la Asunción et al. (2016), which results in a very high efficiency.

### L-HySEA input parameters

**Fig. 3:**
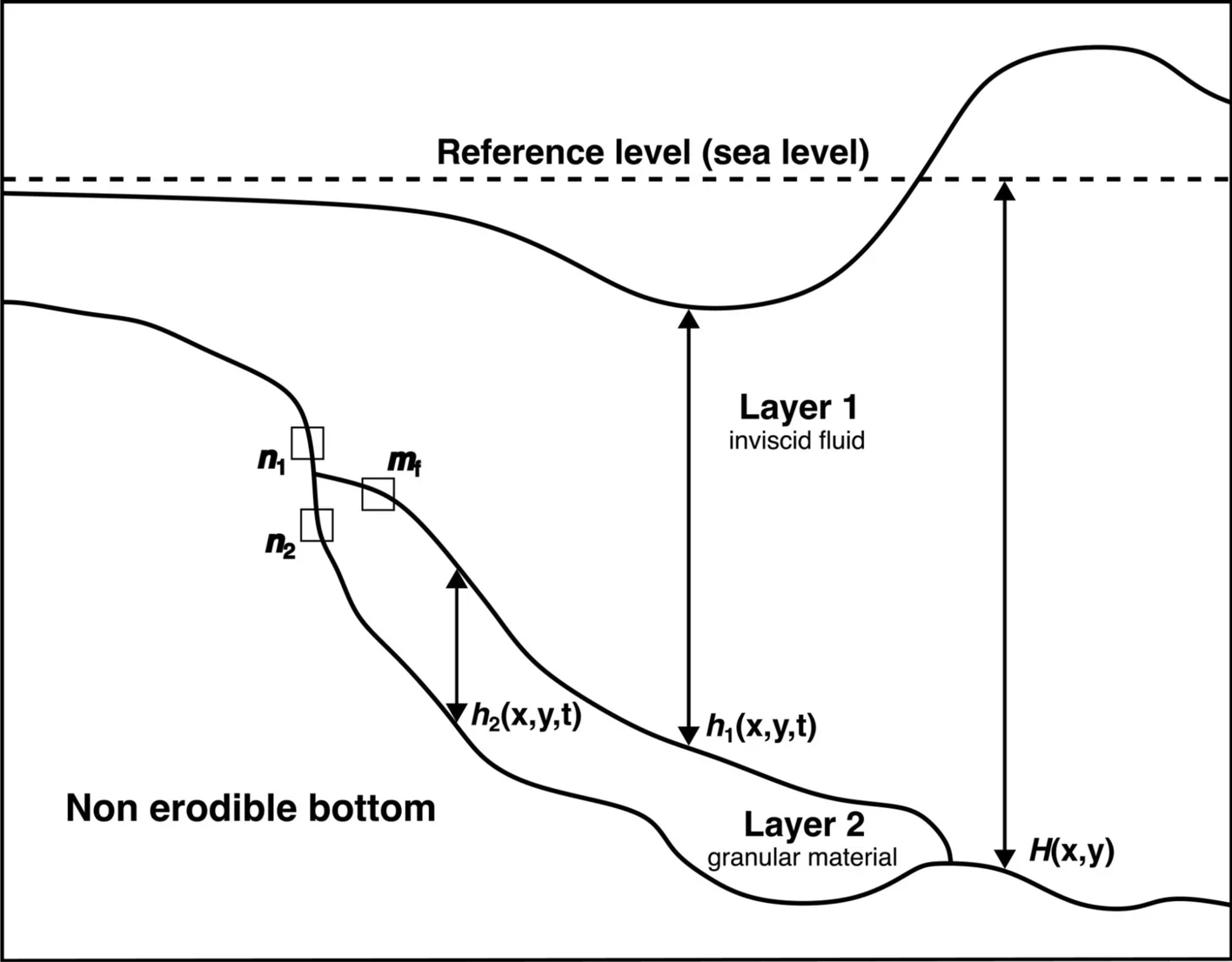
Overview of main system variables and parameters. Non-erodible topography H(x,y), initial thickness of each layer ${h}_{i}(x,y,t)$, friction between layers ${m}_{f}$, friction between water and non-erodible bottom ${n}_{1}$, friction between slide and non-erodible bottom ${n}_{2}$. Modified after Macías et al. (2015)

Input parameters for the L-HySEA code include the non-erodible topography H(x,y), initial thickness of each layer ${h}_{i}(x,y,{t}_{0})$, Coulomb friction angle α, ratio of densities r, friction between layers ${m}_{f}$, friction between water and non-erodible bottom ${n}_{1}$ as well as friction between slide and non-erodible bottom ${n}_{2}$. An overview of main system variables and parameters, except the Coulomb friction angle α and ratio of densities r is presented in Fig. 3. Other input parameters are the total simulated time, time interval for data output, Courant–Friedrichs–Lewy condition as factor between 0 and 1 and maximum velocity for each layer.

González-Vida et al. (2019) previously used the L-HySEA code for the Lituya Bay landslide-generated tsunami. The best parameter setting found by the authors to recreate the Lituya Bay tsunami is a Coulomb friction angle α=13°, ratio of densities r=0.44, which corresponds to a slide layer density ${\rho}_{2}=2275$ kg/m^3^, friction between layers ${m}_{f}=0.08$ and standard values for Manning coefficients ${n}_{1}=0.02$, ${n}_{2}=0.05$ (Arcement et al. 1989; Phillips et al. 2006).

The tsunami impact of a potential Kameni flank collapse is analyzed for all scenarios with the Coulomb friction angle varied between values of α=9,12,15(°), which are well within the range of values described as reasonable by González-Vida et al. (2019). For lava flows, which are assumed to be the sliding layer, the density is varied within values of ${\rho}_{2}= 2700, 2500, 2300$ (kg/m^3^), based on previous studies (Nomikou et al. 2014a, b; Pyle et al. 2006). Due to its much smaller impact on the resulting tsunami, compared to α and ${\rho}_{2}$, the friction between layers is set to ${m}_{f}=0.08$ for all simulations, which is the best fit found by González-Vida et al. (2019) for recreating the Lituya Bay tsunami. For Manning coefficients, standard values ${n}_{1}=0.02$, ${n}_{2}=0.05$ (Arcement et al. 1989; Phillips et al. 2006) are used for all simulations. A topography grid with a resolution of 20 m is used for numerical modeling. Slide planes are created by interpolating from the bottom of the topographic step through Kameni lava flows to the surface of Kameni volcano, which results in slide volumes of 0.057 km^3^, 0.077 km^3^, and 0.13 km^3^ for slides A, B, and C, respectively. A detailed view of the slide planes is shown in the supplementary material (Fig. S1).

### Seafloor microbathymetry

During cruise M215, the Modular Mobility (MOMO) photogrammetric tow-sled system was flown on a transect (dive Mo19) across the northern front of the 1570–1573 flow facing toward the caldera floor (Fig. 1). Maintaining an altitude between 2.5 m and 5 m above the seafloor at a vehicle speed of 0.25 kn, its 16 Mp square sensor stereoscopic cameras with a 100° rectilinear field of view collected longitudinally and laterally highly overlapping seafloor imagery at a 1 s interval. The imagery was consequently processed into a hydroacoustically georeferenced photogrammetric 3D seafloor model using Agisoft Metashape Professional 2.2.1 (cf. Kwasnitschka et al. 2013). The resulting model of 395 m by 93 m extent has a geometric and textural resolution of 1 cm at 9 m average track width.

## Results

### L-HySEA modeling

**Fig. 4:**
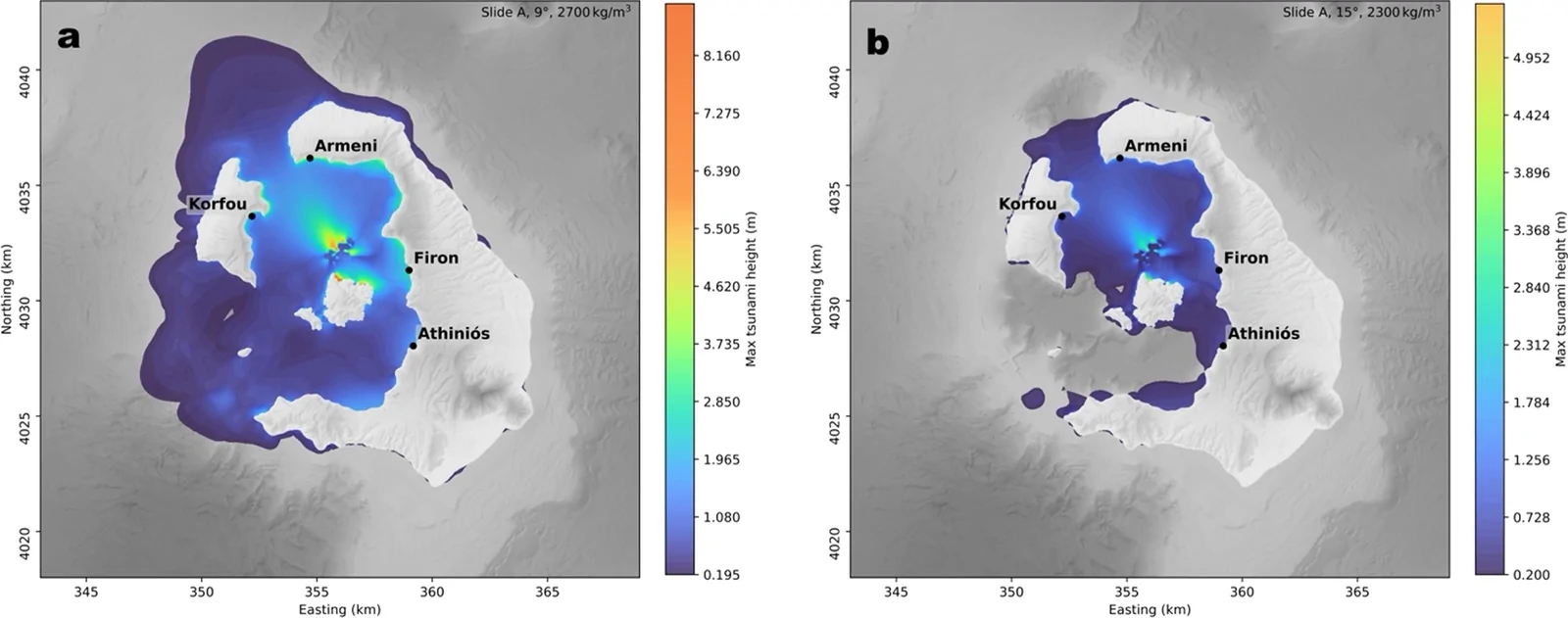
Potential tsunamis slide A. Modeling results of maximum tsunami height (offshore) and flow depth (onshore) for different values of Coulomb friction angle α and slide layer density ${\rho}_{2}$. a) α=9°, ${\rho}_{2}=2700$ kg/m^3^ and b α=15°, ${\rho}_{2}=2300$ kg/m^3^

**Fig. 5:**
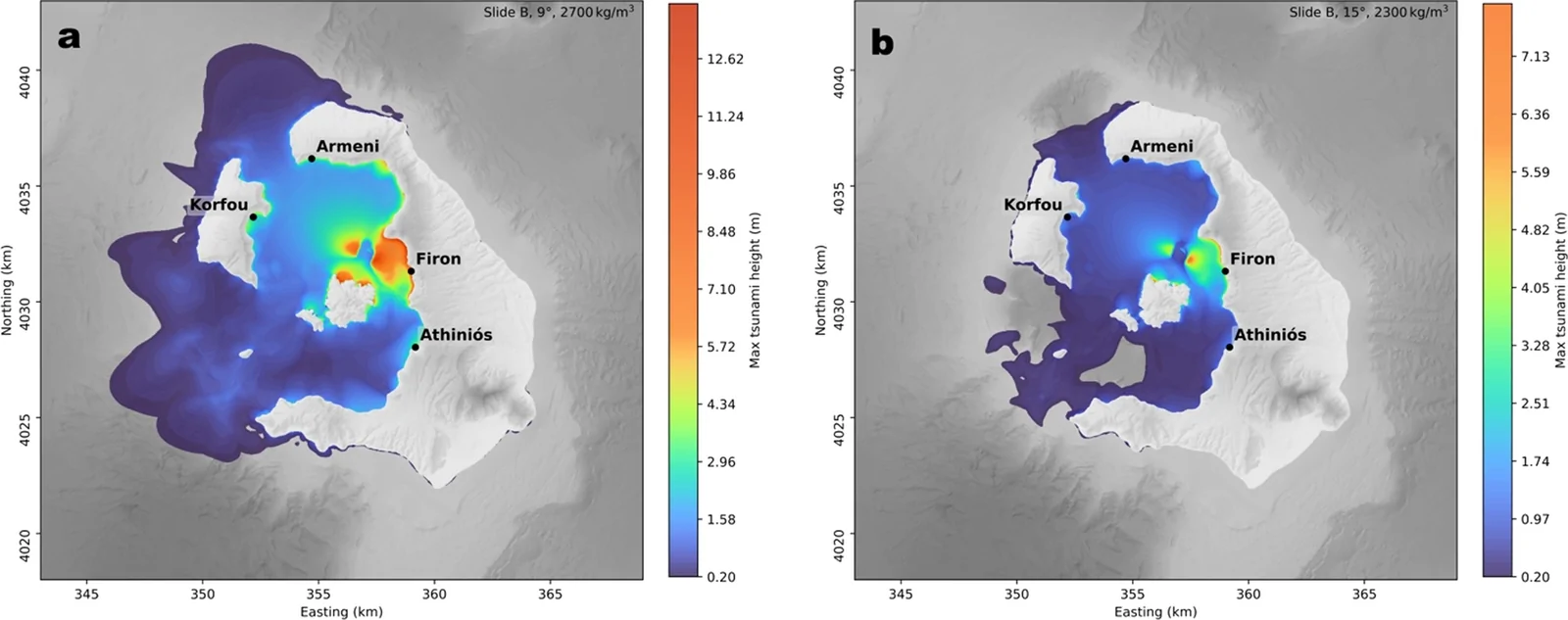
Potential tsunamis slide B. Modeling results of maximum tsunami height (offshore) and flow depth (onshore) for different values of Coulomb friction angle α and slide layer density ${\rho}_{2}$. **a** α=9°, ${\rho}_{2}=2700$ kg/m^3^ and **b** α=15°, ${\rho}_{2}=2300$ kg/m^3^

**Fig. 6:**
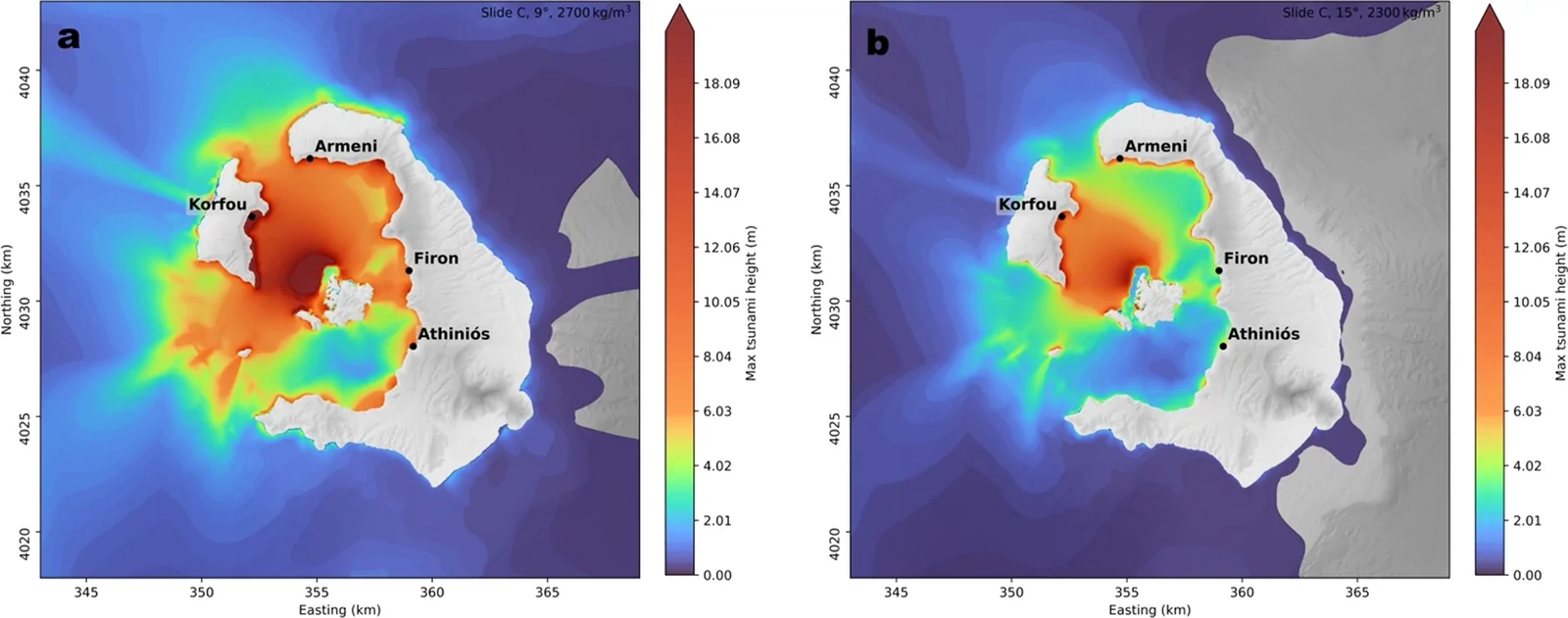
Potential tsunamis slide C. Modeling results of maximum tsunami height (offshore) and flow depth (onshore) for different values of Coulomb friction angle α and slide layer density ${\rho}_{2}$. **a** α=9°, ${\rho}_{2}=2700$ kg/m^3^ and **b** α=15°, ${\rho}_{2}=2300$ kg/m^3^

**Fig. 7:**
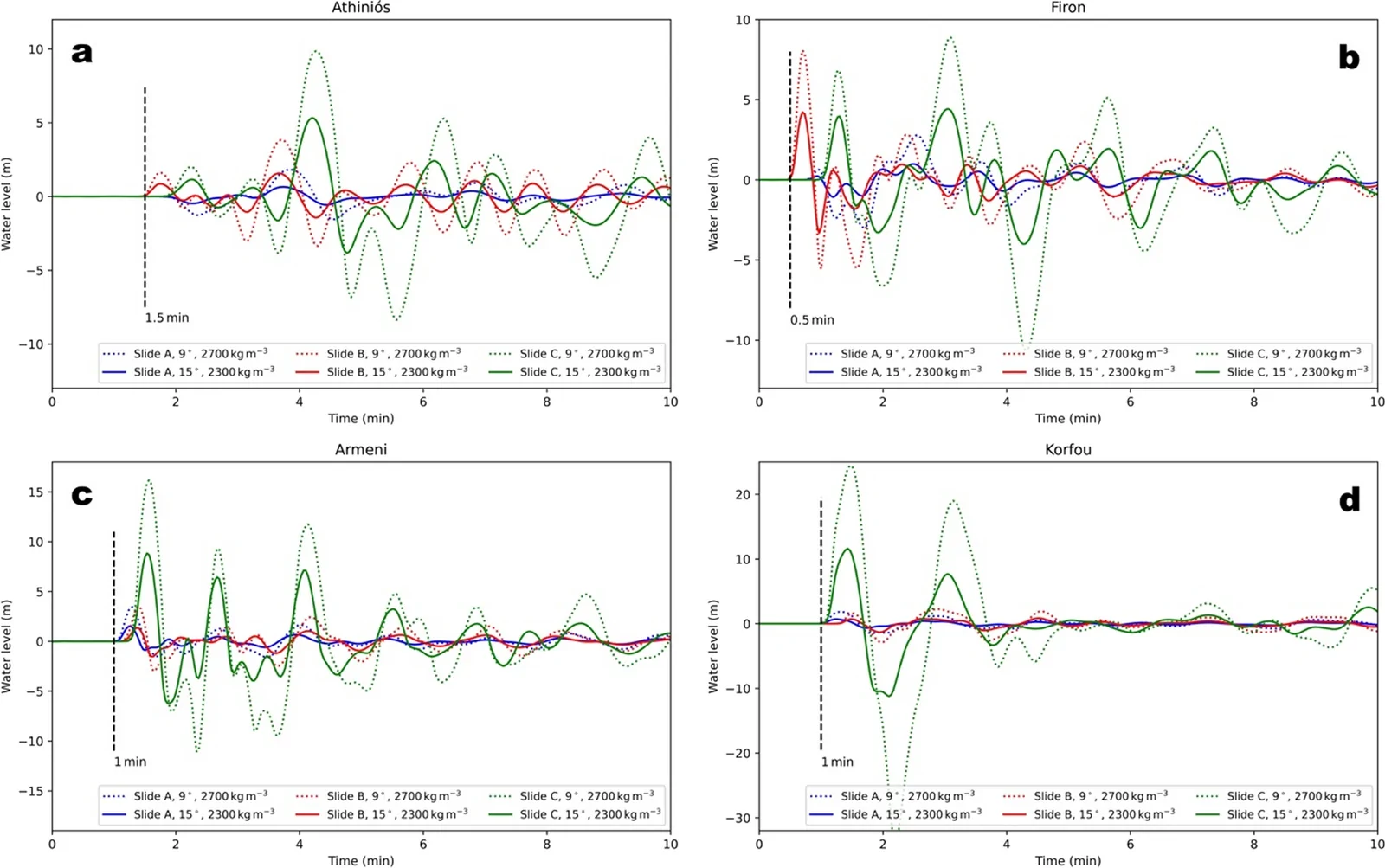
Virtual tide gages. Modeling results of water level at four selected locations (i.e., Korfou, Armeni, Firon, Athiniós). Tide gages for slide A, B, C are shown in blue, red, green, respectively. Solid lines are the results for α=15°, ${\rho}_{2}=2300$ kg/m^3^ and dotted lines for α=9°, ${\rho}_{2}=2700$ kg/m^3^

In Figs. 4, 5, and 6, the maximum tsunami height (offshore) and the flow depth (onshore) at each grid point are shown for slides A, B, and C, respectively. The modeling results strongly depend on the values of two key input parameters: The Coulomb friction angle α and the sliding layer density ${\rho}_{2}$. For all slides, the two combinations of α and ${\rho}_{2}$ with the highest and the lowest resulting tsunami within the considered parameter range are shown in the main text (i.e., α = 9°, ${\rho}_{2}$= 2700 kg/m^3^ and α = 15°, ${\rho}_{2}$= 2300 km/m^3^, respectively). All results are shown with a shared color-scale and labels adjusted to the maximum tsunami height of each parameter combination. All other results are shown in the supplementary material (Figs. S2, S3, S4). Virtual tide gages for all slides at the four selected locations (i.e., Korfou, Armeni, Firon, Athiniós) are presented in Fig. 7.

Necmioglu et al. (2023) reported maximum tsunami amplitudes and arrival times in the order of up to 60 m and 1–2 min for some locations at the Santorini Island, which is in good agreement with our results: The resulting tsunami for the scenario of slide A (Fig. 4) is NW-directed but with a smaller maximum height between 2 and 4 m near Armeni. The first arrival at Armeni is 1 min after the slide motion is initiated. For the scenario of slide B (Fig. 5), the resulting tsunami runs in a NE-direction with a maximum height between 7 and 13 m just north of the Firon. The first arrival at Firon is less than 30 s after the slide motion is initiated. The resulting tsunami for the scenario of slide C (Fig. 6) is NW-directed, with a maximum height between 21 and 39 m at Korfou, and the first arrival at Korfou is 1 min after the slide motion is initiated (Table 1). For each scenario, the semi-confined geometry of the caldera would promote tsunami reflections, causing multiple impacts at individual coastal locations within the first minutes (Fig. 7).

**Table 1:** Summary of modeling results

Scenario	Direction	Maximum height	First arrival	Location of max height / first arrival
A	NW	2 m–4 m	~60 s	Armeni
B	NE	7 m–13 m	< 30 s	Firon
C	NW	21 m–39 m	~60 s	Korfou

For all three scenarios, the chosen step of $\Delta \alpha =3$° has a stronger impact on the resulting tsunami than the chosen step of ${\Delta \rho }_{2}=200$ kg/m^3^, and, as intuitively expected, the maximum tsunami height correlates with the slide volume.

### Seafloor microbathymetry

**Fig. 8:**
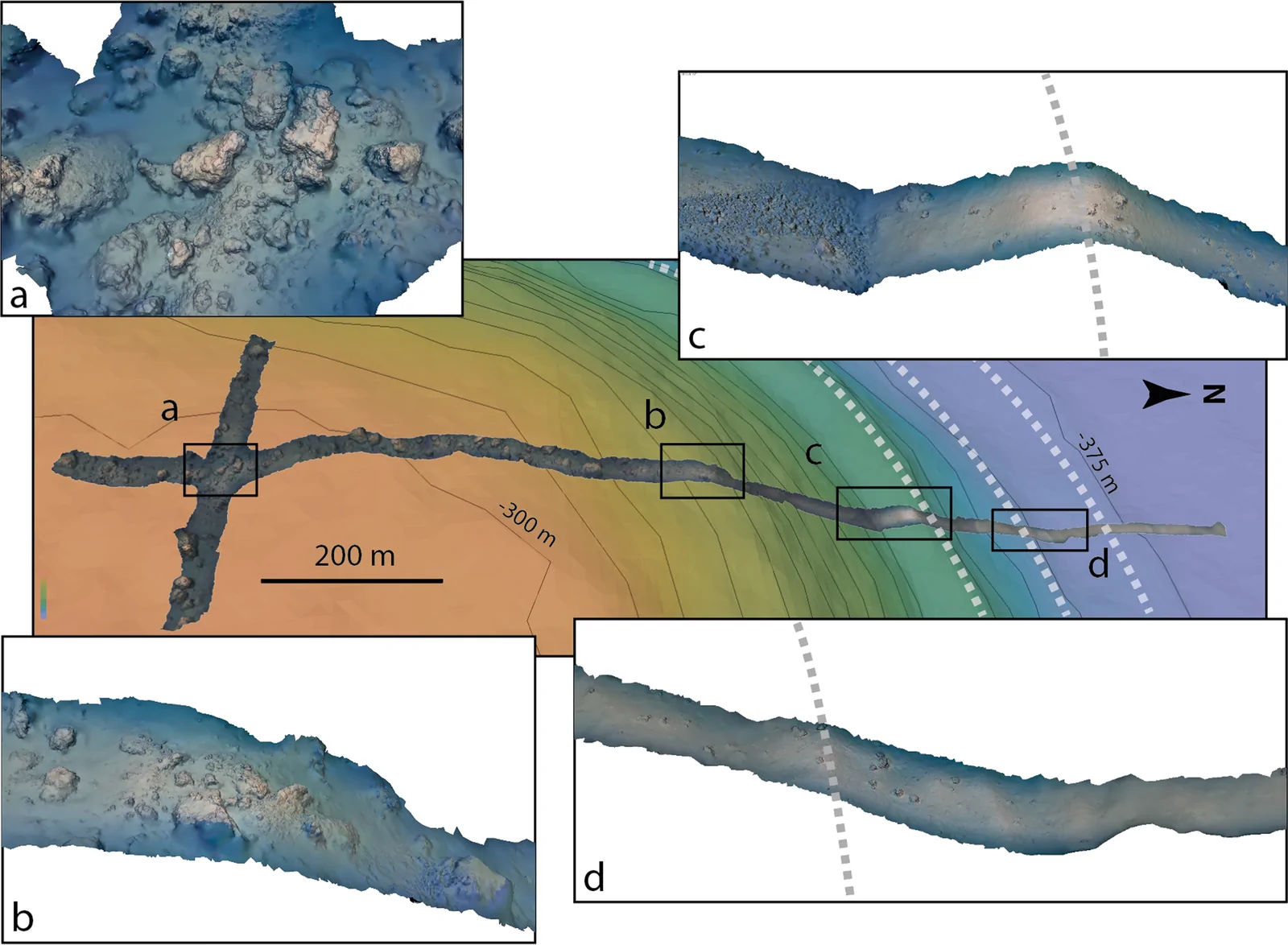
Oblique views of sections of the photogrammetric terrain model created from MOMO tow-sled imagery across the frontal slope of the 1570–76 flow. **a** shows brecciated lava boulders of up to 3 m diameter, **b** marks the crest for the frontal slope while **c** shows its foothill and, together with **d**), a series of moraines of marine background sediment created by the burrowing lava flow. Dashed lines in all subfigures indicate moraines. The background bathymetry is derived from 10 m-resolution M215 EM710 multibeam mapping. For reference of scale, the average track width is 9 ±2 m

The photogrammetric model reveals that the lava flow morphology is a direct continuation of the upper, subaerial morphology (Fig. 8). Brecciated boulders of up to 5 m diameter are scattered across the main axis of flow lobes, covering about 50% of the surface. Toward the crest and the frontal slope, both coverage and diameter decrease to a maximum of 2 m diameter and 20% coverage. The frontal slope of the flow is covered by moderately well sorted talus of lava breccia up to 20 cm in diameter, forming individual sedimentary fans suggesting recent redeposition in contrast to surrounding, slightly sedimented areas. Less than 5% of individual larger boulders up to 50 cm cover the lower foothill that burrows into the bright clay covering the caldera floor, creating a series of moraines spaced 35 m apart visible as morphological steps with a height of 2 m, 3 m, and 7 m toward the flow, with an ever sharpening relief. There were no signs of recent deformation, e.g., cracks or fissures.

## Discussion

### Hazard assessment for modeled tsunamis

**Fig. 9:**
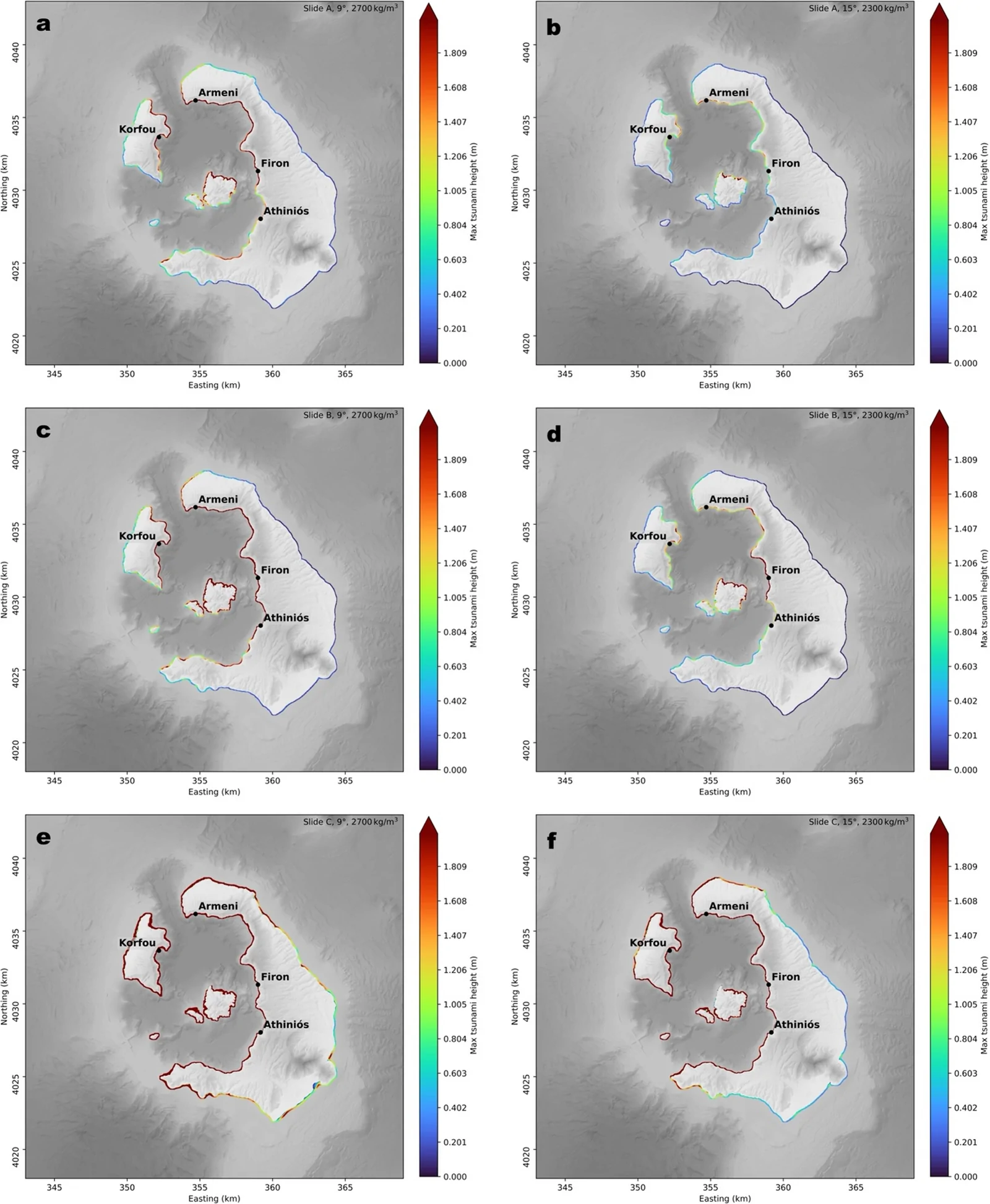
Coastal impact **a**, **b** slide A, **c**, **d** slide B, and **e**, **f** slide C. Modeling results of maximum tsunami height along the coastline for different values of Coulomb friction angle α and slide layer density${\rho}_{2}$. **a**, **c**, **e** α=9°, ${\rho}_{2}=2700$ kg/m^3^ and **b**, **d**, **f** α=15°, ${\rho}_{2}=2300$ kg/m^3^. Color-scale limited to 2 m

The numerical modeling performed in our study is a first-order approximation and the resulting tsunami height of all scenarios nearly doubles within the considered parameter range. Yet, numerical modeling shows that all three potential slides can reach coastal tsunami heights exceeding 2 m at several critical locations within Santorini’s caldera (Fig. 9). It should be noted that the color-scales in Fig. 9 are limited to 2 m, but especially slides B and C reach coastal tsunami heights much higher than 2 m, as also shown in the virtual tide gages (Fig. 7). Such high tsunami amplitudes are in agreement with a previous study by Necmioglu et al. (2023), in which possible landslide scenarios at Santorini are simulated through a Gaussian shaped initial wave.

### Exposure and vulnerability of maritime infrastructure

A tsunami wave exceeding 2 m would have a significant impact at all four critical locations, where infrastructure is at sea level or elevated by just about 1 m. The small harbor and the beach at Korfou are visited by thousands of tourists during summer peaks. While the town of Oia sits atop steep cliffs, its maritime lifeline, the critical infrastructure at Armeni, lies at the bottom. Similarly, Fira’s old harbor remains a high-density tourist node. Most critically, the Athiniós ferry port serves as Santorini’s sole terminal for large vessels, handling an average of 15 to 25 ferry arrivals daily during the high season. Beyond passenger transit, Athiniós is the exclusive gateway for 100% of the island’s essential imports, including freshwater tankers, groceries, and fuel. A tsunami-induced disruption of this single terminal would immediately paralyze the supply chain (e.g., through damage to the access road, vessels, or moorings). Given that the island maintains limited storage for perishables and water, even a 48 to 72 h port closure would lead to critical shortages of basic supplies for both the 25,000 permanent residents and the roughly 50,000 daily visitors. Consequently, the vulnerability of Athiniós represents a systemic risk to the island’s functional continuity. It should be noted that the number of tourists varies strongly throughout the year, peaking during the summer months with daily visitors easily doubling the resident population on the island. During the winter season, international direct flights cease, most resorts and businesses shut down, and daily visitor presence drops significantly compared to the permanent population. However, the island’s dependence on Athiniós as its exclusive gateway for essential goods persists throughout the year, regardless of the time of year.

In addition to coastal infrastructure, large tsunami waves would also have an impact on cruise ships as well as small boats, which transfer tourists between cruise ships and e.g., Fira. Transfer operations would likely have to be suspended, preventing thousands of cruise ship passengers from reaching or returning from shore, even without substantial damage to harbor infrastructure or the cruise ships. Recent data from the Hellenic Ports Association (2024) indicate that Santorini now hosts approximately 800 cruise ship calls annually, with passenger arrivals exceeding 1.2 million. During peak periods, daily visitor influxes at sea-level embarkation points, specifically the high-traffic nodes of Athiniós and the Old Harbor of Fira, reach 18,000 individuals. Given that critical maritime infrastructure and passenger terminals are situated at elevations of approximately 1 m above mean sea level, the population at risk during a tsunamigenic event is substantial.

### Lavaflow emplacement and possible remobilization

The MOMO survey revealed that at least the 1570–1573 lavaflow was compact enough to burrow into the sediment over a distance of at least 50 m as it crept downslope, judging by the transverse ridges it created. The absence of fresh surficial tectonism suggests there is little remobilization despite ongoing volcano–seismic activity. Hürlimann et al. (2000) investigated the mechanical relationship between caldera collapse events and submarine landslides. The authors conclude that seismic ground acceleration, related to nearby, shallow earthquakes (Mw > 5) caused by caldera collapses, can trigger slope failures. Although the geological setting considered by Hürlimann et al. (2000) differs from that examined here, Santorini is an active volcano–tectonic region. The surrounding area has experienced major tsunamigenic earthquakes, including the devastating 1956 events (Mw 7.5 and 7.2; Papadopoulos et al. 1992; Okal et al. 2009; Brüstle et al. 2014), and strong seismicity was also recorded during the 2025 Kolumbo dyke intrusion (Isken et al. 2025). These observations indicate that future earthquakes of similar magnitude are plausible and could trigger submarine landslides through seismic ground acceleration.

## Conclusion

Owing to the position of the Kameni volcanic edifice atop a northward-deepening topographic step coincident with the volcano–tectonic Kameni Line, previous research has identified a susceptibility to flank instability (Preine et al. 2024). Numerical simulations of multiple potential flank-collapse scenarios reveal a significant tsunamigenic hazard, utilizing a parametric range consistent with the methodology of González-Vida et al. (2019). Specifically, a localized failure (Slide B) involving a volume of 0.13 km^3^ could generate maximum tsunami amplitudes of up to 39 m. Furthermore, all modeled scenarios, with landslide volumes ranging from 0.057 km^3^ to 0.13 km^3^, result in wave heights exceeding 2 m at several primary ports, including the high-traffic sites of Korfou and Armeni, the historic harbor of Fira, and the Athiniós ferry terminal.

Given that the Santorini volcanic complex resides within one of the most geodynamically active and hazardous volcano–tectonic zones in the Mediterranean (Druitt et al. 1999, 2019; Preine et al. 2026), seismically induced slope failures represent a plausible and significant tsunami trigger. The depth-averaged numerical modeling presented here provides a first-order hazard assessment for the three identified landslide scenarios. To refine these results for high-density population centers and critical infrastructure, future research employing full three-dimensional (3D) hydrodynamic models is recommended, though such high-resolution modeling remains beyond the scope of the current study.

## Supplementary Information

Below is the link to the electronic supplementary material.

Supplementary file1 (DOCX 1719 KB)

## Data Availability

This study uses openly available EMODnet and PROTEUS (MGL1521) data, which can be found on the EMODnet bathymetry portal at https://portal.emodnet-bathymetry.eu and https://www.marine-geo.org/tools/files/31252#datasets, respectively.
