# Supplementary material for: Tsunamigenic potential of flank instabilities of Kameni Volcano inside Santorini Caldera, southern Aegean
Source: Int J Earth Sci. 2026 Oct 21;115(6):73. doi: 10.1007/s00531-026-02623-5 (PMC13687423; doi:10.1007/s00531-026-02623-5)
Supplement: Supplementary file 1 [file 531_2026_2623_MOESM1_ESM.docx]

## Supplementary Information

**Fig. S1** Kameni slide planes. Slide plane (a) and true topography (b) for all three potential flank collapse scenarios A, B, C (blue, red, green, respectively).

**Fig. S2** Potential tsunamis slide A. Modeling results of maximum tsunami height (offshore) and flow depth (onshore) for different values of Coulomb friction angle $\alpha$ and slide layer density $\rho_{2}$. Coulomb friction angle $\alpha=9, 12, 15$(°) from top to bottom, respectively. Slide layer density $\rho_{2}=2700, 2500, 2300$ (kg/m^3^) from left to right, respectively.

**Fig. S3** Potential tsunamis slide B. Modeling results of maximum tsunami height (offshore) and flow depth (onshore) for different values of Coulomb friction angle $\alpha$ and slide layer density $\rho_{2}$. Coulomb friction angle $\alpha=9, 12, 15$(°) from top to bottom, respectively. Slide layer density $\rho_{2}=2700, 2500, 2300$ (kg/m^3^) from left to right, respectively.

**Fig. S4** Potential tsunamis slide C. Modeling results of maximum tsunami height (offshore) and flow depth (onshore) for different values of Coulomb friction angle $\alpha$ and slide layer density $\rho_{2}$. Coulomb friction angle $\alpha=9, 12, 15$(°) from top to bottom, respectively. Slide layer density $\rho_{2}=2700, 2500, 2300$ (kg/m^3^) from left to right, respectively.
